# Impact of direct mesenteric perfusion on malperfusion in acute type A aortic dissection repair

**DOI:** 10.1093/ejcts/ezae452

**Published:** 2024-12-16

**Authors:** Ryota Yamamoto, Wataru Kato, Yoshiyuki Tokuda, Koshi Yamaki, Koji Morita, Tomonari Uemura, Toshikuni Yamamoto, Hideki Ito, Tomo Yoshizumi, Sachie Terazawa, Yuji Narita, Masato Mutsuga

**Affiliations:** Department of Cardiac Surgery, Nagoya University Graduate School of Medicine, Nagoya, Japan; Department of Cardiovascular Surgery, Japanese Red Cross Aichi Medical Center Nagoya Daini Hospital, Nagoya, Japan; Department of Cardiac Surgery, Nagoya University Graduate School of Medicine, Nagoya, Japan; Department of Cardiovascular Surgery, Japanese Red Cross Aichi Medical Center Nagoya Daini Hospital, Nagoya, Japan; Department of Cardiovascular Surgery, Japanese Red Cross Aichi Medical Center Nagoya Daini Hospital, Nagoya, Japan; Department of Cardiac Surgery, Nagoya University Graduate School of Medicine, Nagoya, Japan; Department of Cardiac Surgery, Nagoya University Graduate School of Medicine, Nagoya, Japan; Department of Cardiac Surgery, Nagoya University Graduate School of Medicine, Nagoya, Japan; Department of Cardiac Surgery, Nagoya University Graduate School of Medicine, Nagoya, Japan; Department of Cardiac Surgery, Nagoya University Graduate School of Medicine, Nagoya, Japan; Department of Cardiac Surgery, Nagoya University Graduate School of Medicine, Nagoya, Japan; Department of Cardiac Surgery, Nagoya University Graduate School of Medicine, Nagoya, Japan

**Keywords:** Aortic dissection, Laparotomy, Mesenteric artery, Malperfusion, Temporary perfusion

## Abstract

**OBJECTIVES:**

Mesenteric malperfusion in acute aortic dissection remains a life-threatening complication with no standardized treatment strategy. This study aimed to describe and evaluate the outcomes of our integrated approach combining exploratory laparotomy, immediate mesenteric reperfusion, and central aortic repair.

**METHODS:**

We retrospectively reviewed patients with acute aortic dissection with a preoperative diagnosis of mesenteric malperfusion who were treated between August 2011 and November 2022. Our surgical approach was to establish cardiopulmonary bypass, followed by exploratory laparotomy with mesenteric artery flow assessment using Doppler ultrasound and direct perfusion if needed, central aortic repair, and subsequent mesenteric artery reconstruction. The primary end-point was the 30-day operative mortality.

**RESULTS:**

Among 217 patients with acute aortic dissection, 12 (5.5%) had mesenteric malperfusion on preoperative computed tomography. Ten patients underwent exploratory laparotomy, where Doppler ultrasonography revealed reduced mesenteric blood flow in five patients (2.3% of the total 217 patients). These patients underwent direct perfusion of the mesenteric artery via a side branch of the cardiopulmonary bypass circuit. Doppler ultrasound confirmed the restoration of mesenteric blood flow in all perfused patients. No bowel resections were required. The operative mortality in patients with mesenteric malperfusion was 20%. The causes of death were stroke (*n* = 1) and acute myocardial infarction (*n* = 1).

**CONCLUSIONS:**

Our integrated surgical strategy combining central aortic repair with concurrent exploratory laparotomy and immediate mesenteric perfusion demonstrated technical feasibility in managing mesenteric malperfusion during aortic repair. Further prospective studies with larger cohorts are warranted to validate these findings.

## GRAPHICAL ABSTRACT

**Figure:**
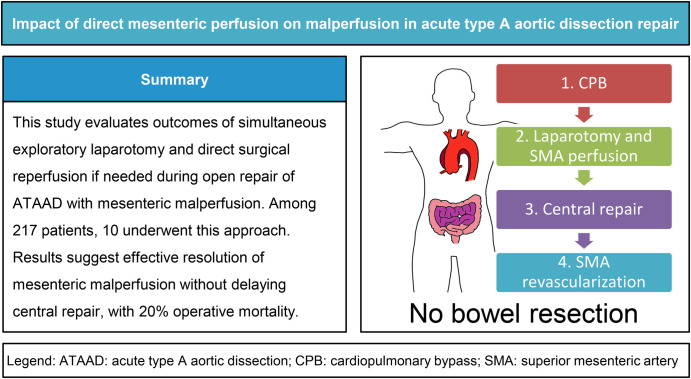

## INTRODUCTION

Despite recent improvements in surgical outcomes for acute type A aortic dissection (ATAAD), the Japanese Association for Thoracic Surgery reported that the in-hospital mortality rate was 11.2% (670/5995) in 2017 [1], with patients with mesenteric malperfusion showing mortality rates greater than 60% [2, 3].

The traditional approach for ATAAD with mesenteric malperfusion has involved immediate central repair followed by bowel resection if needed. This is because many surgeons believe that the best strategy for treating malperfusion syndrome is to rapidly improve flow to the true lumen and reduce pressure in the false lumen. However, central repair can be time-consuming and may not adequately address branch-type static obstructions of the superior mesenteric artery (SMA) [4]. In addition, even when bowel resection is performed, the results may be disastrous.

Recent reports suggest that treating intestinal ischaemia before central repair may improve the outcomes [5, 6]. However, this is impossible in unstable patients. Furthermore, a certain number of deaths due to aortic rupture have been reported when surgeons have prioritized revascularization of the mesenteric artery through endovascular treatment [7]. As both intestinal ischaemia and aortic dissection often undergo various clinical transitions, the optimal management remains unclarified. We consider it important to quickly resolve these two problems.

Since 2011, the Japanese Red Cross Aichi Medical Center Nagoya Daini Hospital has used a specific approach for mesenteric malperfusion in ATAAD based on preoperative CT scans. While temporary SMA perfusion using saphenous vein bypass grafting during ATAAD repair was first reported by Okada *et al.* in 2007 [8], our approach involves exploratory laparotomy and immediate surgical reperfusion with SMA plasty concurrent with open repair of ATAAD. The present study aimed to describe our systematic surgical approach for concurrent management of central aortic repair and mesenteric malperfusion, and to report the clinical outcomes of this integrated strategy in patients with and without temporary SMA perfusion.

## PATIENTS AND METHODS

This retrospective observational study examined consecutive patients who underwent repair of ATAAD between August 2011 and November 2022. ATAAD was defined as an onset within 14 days of admission. Patients were included if they had suspected mesenteric malperfusion based on preoperative CT findings showing SMA dissection and clinical symptoms such as abdominal pain and tenderness. Patients were excluded if they had pre-existing bowel necrosis at laparotomy precluding central repair, or if SMA revascularization was performed separately from central repair. The primary end-point was the 30-day operative mortality. The secondary end-points were successful SMA revascularization (confirmed by postoperative contrast CT), need for bowel resection, laparotomy-related complications (surgical site infection, prolonged ileus, and reoperation), and aortic event-free survival. Aortic events were defined as aortic reoperation, aortic rupture, or aortic-related death. The Institutional Review Board of the Japanese Red Cross Aichi Medical Center Nagoya Daini Hospital approved the study (approval no. 1632) on 6 September 2024 and waived the need for consent.

### Diagnosis of mesenteric malperfusion

Malperfusion was defined as a failure of blood flow to end organs caused by dissection-related obstruction of the aorta and its branches. Based on contrast-enhanced CT findings and clinical assessment, patients with aortic dissection extending into the SMA were diagnosed with mesenteric malperfusion. The diagnostic criterion for mesenteric malperfusion was either: true lumen narrowing of the SMA on CT with clinical symptoms (abdominal pain, hematochezia) or complete occlusion of the SMA true lumen on CT, regardless of symptoms. This classification was maintained regardless of subsequent intraoperative findings after cardiopulmonary bypass (CPB) initiation. In all patients meeting these diagnostic criteria, we performed laparotomy and SMA perfusion simultaneously with CPB establishment.

### Operative technique

After being diagnosed with ATAAD, patients were expeditiously transferred to the operating theater for emergency repair. The procedure was performed under general anaesthesia, with the patients intubated and in the supine position. All procedures, including SMA access and reconstruction, were performed by cardiac surgeons experienced in aortic surgery. Additional surgical specialists were not required.

#### Surgical approach

The initial phase involved exposing the undissected right axillary and common femoral arteries for arterial access. A median sternotomy incision was extended to the umbilicus to expose the SMA. This manoeuvre typically alleviated shock due to cardiac tamponade. The right atrium was exposed for venous access.

#### Anticoagulation and CPB

Heparin (300 U/kg) was administered to maintain an activated clotting time of longer than 400 seconds. CPB was established using right axillary and femoral arterial cannulation for inflow and right atrial drainage for outflow. A left ventricular vent was inserted through the right superior pulmonary vein, followed by systemic cooling to the target temperature. No endotoxin absorption was used during CPB.

#### SMA management

**Figure 1:**
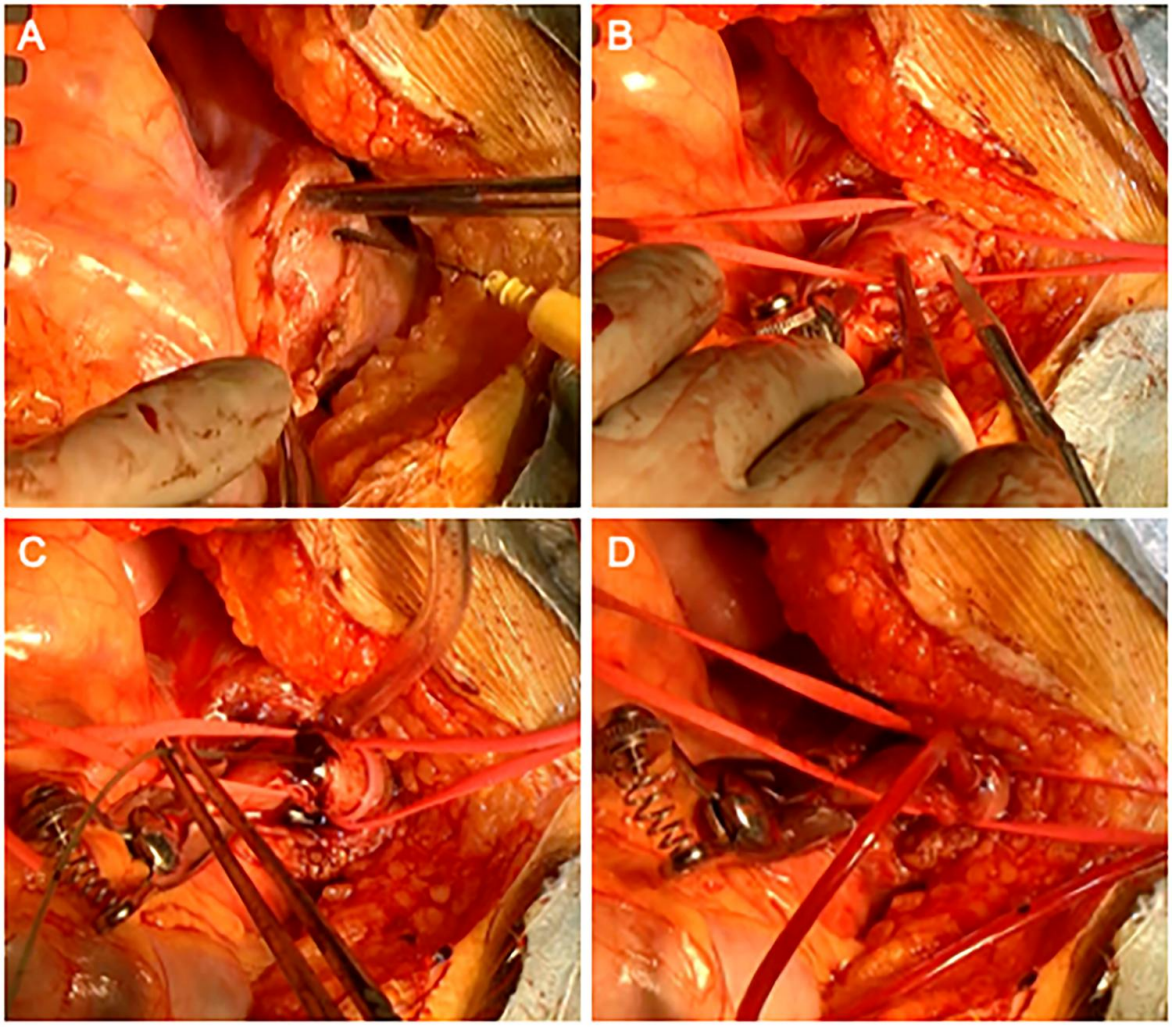
Surgical procedures in the superior mesenteric artery (SMA) perfusion. (**A**) Exposure of the SMA through a transverse mesenteric incision. (**B**) The SMA after taping and clamping, with a transverse incision. (**C**) Removal of any substantial thrombi from the false lumen using a 4-French balloon catheter. (**D**) Insertion of an 8-French soft tube into the peripheral true lumen, connected to a side branch of the cardiopulmonary bypass circuit for perfusion.

**Figure 2:**
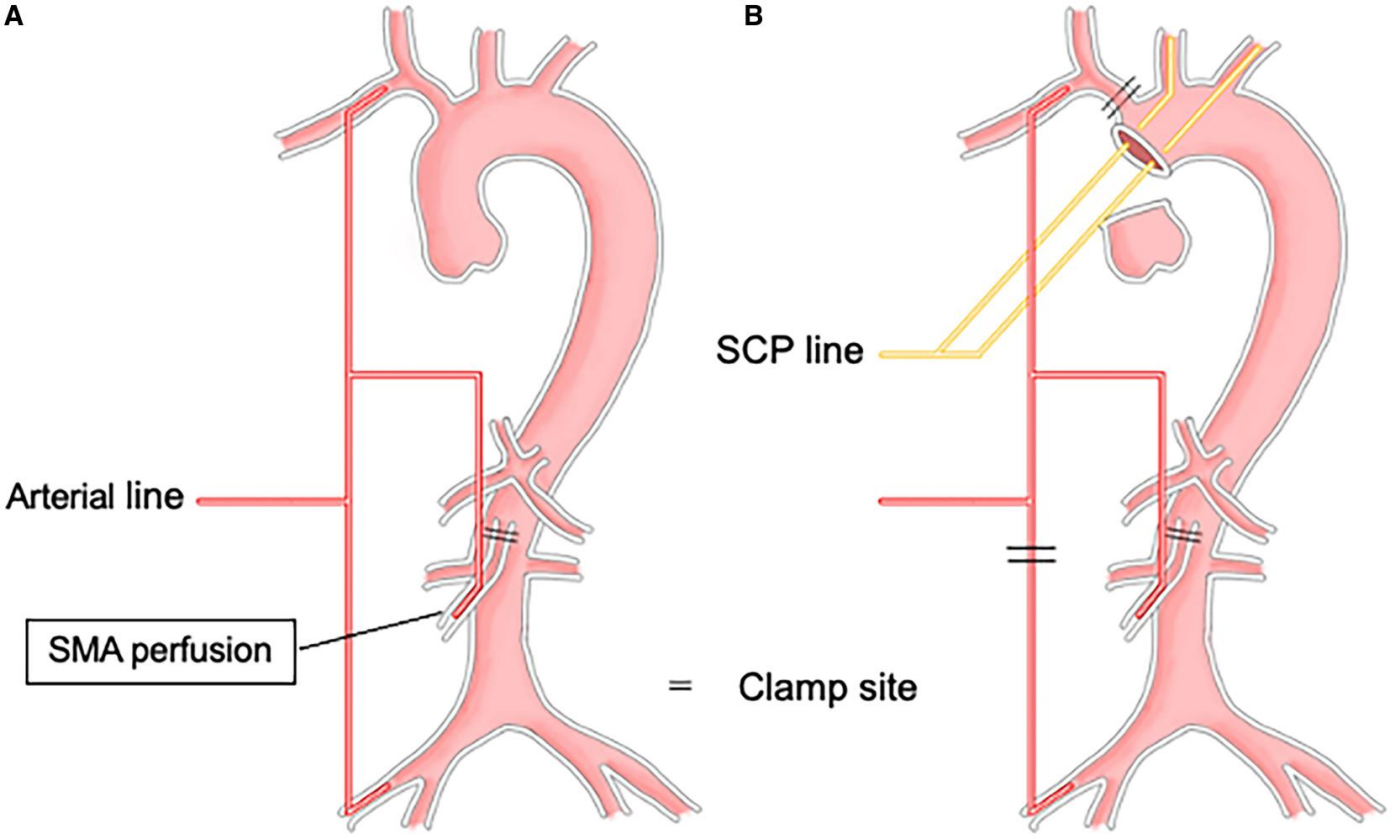
Schematic drawing of the SMA perfusion during cardiopulmonary bypass. (**A**) The SMA perfusion route is connected to a side branch of the axillary artery cannulation line. (**B**) During circulatory arrest, the femoral perfusion and brachiocephalic artery are clamped. SMA perfusion occurs concurrently with right common carotid artery perfusion. SMA: superior mesenteric artery.

Concurrent with systemic cooling, an upper median laparotomy was performed. The transverse colon was manually elevated, and the mesentery was incised to expose the main SMA trunk (Fig. 1A). As demonstrated in Video 1, the SMA was taped distally beyond the middle colic artery branching site. If the intestine showed signs of compromised viability, such as discoloration or reduced peristalsis, and Doppler ultrasound (Model 811-B, Parks Medical) detected reduced SMA flow, SMA perfusion was initiated. The SMA was clamped and transversely incised (Fig. 1B). When a false lumen thrombus was observed upon incision of the SMA, thrombectomy was performed using a 4-French balloon catheter (Fig. 1C). The thrombectomy prioritized perfusion over complete removal, focusing only on achieving adequate space for the perfusion tube and intimal fixation. If resistance was felt, to prevent a new tear, catheter insertion was withheld and the balloon expansion was limited to the thickness of the short axis of the false lumen. Subsequently, an 8-French multipurpose tube (Atom Medical Corp.) was inserted into the distal true lumen of the SMA (Fig. 1D) and connected to the CPB arterial line branch (Fig. 2A). The cannula to the SMA was connected to the main arterial line of the CPB circuit, with no independent flow regulation to the SMA. The mesenteric perfusion was therefore dependent on the pressure from the main arterial line. The bowel was observed for improvements in colour and peristalsis, with Doppler ultrasound repeated if necessary to confirm the effectiveness of mesenteric perfusion.

#### Central aortic repair

Following establishment of adequate SMA perfusion, central aortic repair was commenced. Distal aortic anastomosis was performed under hypothermic circulatory arrest with selective cerebral perfusion at a bladder temperature of 25°C. During circulatory arrest, the brachiocephalic artery was clamped and axillary perfusion was initiated to ensure blood flow to the right common carotid artery and SMA (Fig. 2B). The initial flow through both the right axillary artery and the SMA was set at 5–8 ml/kg/min, and then adjusted up to 15 ml/kg/min according to the perfusion pressure required to maintain adequate perfusion.

#### Selective cerebral perfusion

The protocol for selective cerebral perfusion aimed to establish flow in all three major vessels. While the right common carotid artery was perfused via axillary cannulation, a separate pump system maintained perfusion of the left common carotid and left subclavian arteries.

#### Extent of aortic replacement

The extent of aortic replacement was determined by the location of the entry tear. Ascending aortic replacement was performed for tears in the ascending aorta, partial arch replacement was performed for proximal arch tears, and total arch replacement with a frozen elephant trunk technique was performed for tears of the distal arch and beyond.

#### SMA revascularization

During rewarming following central repair, SMA revascularization (SMA plasty) was conducted as previously described [9]. In cases of persistent inadequate proximal SMA blood flow or suspected static occlusion/stenosis, thrombectomy of both the pseudo and true lumens was performed using a balloon-tip catheter. Distal SMA thrombectomy followed a similar procedure. Upon improvement of blood flow from both sides, the pseudo lumen of the distal SMA was closed with a running 7-0 polypropylene suture connecting the intima and adventitia. The reconstructed distal SMA wall and proximal adventitia were then anastomosed with 7-0 polypropylene. Post-anastomosis Doppler flowmetry was performed to confirm SMA blood flow.

#### Final assessment

The procedure concluded with a meticulous assessment of the bowel coloration and peristalsis. Upon confirmation of adequate perfusion, the abdomen was closed. Lactate monitoring consisted of a preoperative measurement at admission, intraoperative measurements every 30–45 min, and postoperative measurements in the ICU every 30 min for three readings, followed by every 2 hours after stabilization.

### Statistical analysis

Given the small sample size, continuous data are expressed as median [interquartile range], while categorical variables are expressed as *n* (%). Due to the limited number of patients, formal statistical comparisons between groups were not performed. Follow-up completeness was assessed using the formal person-time method. Aortic event-free survival was estimated using the Kaplan–Meier method.

## RESULTS

**Figure 3:**
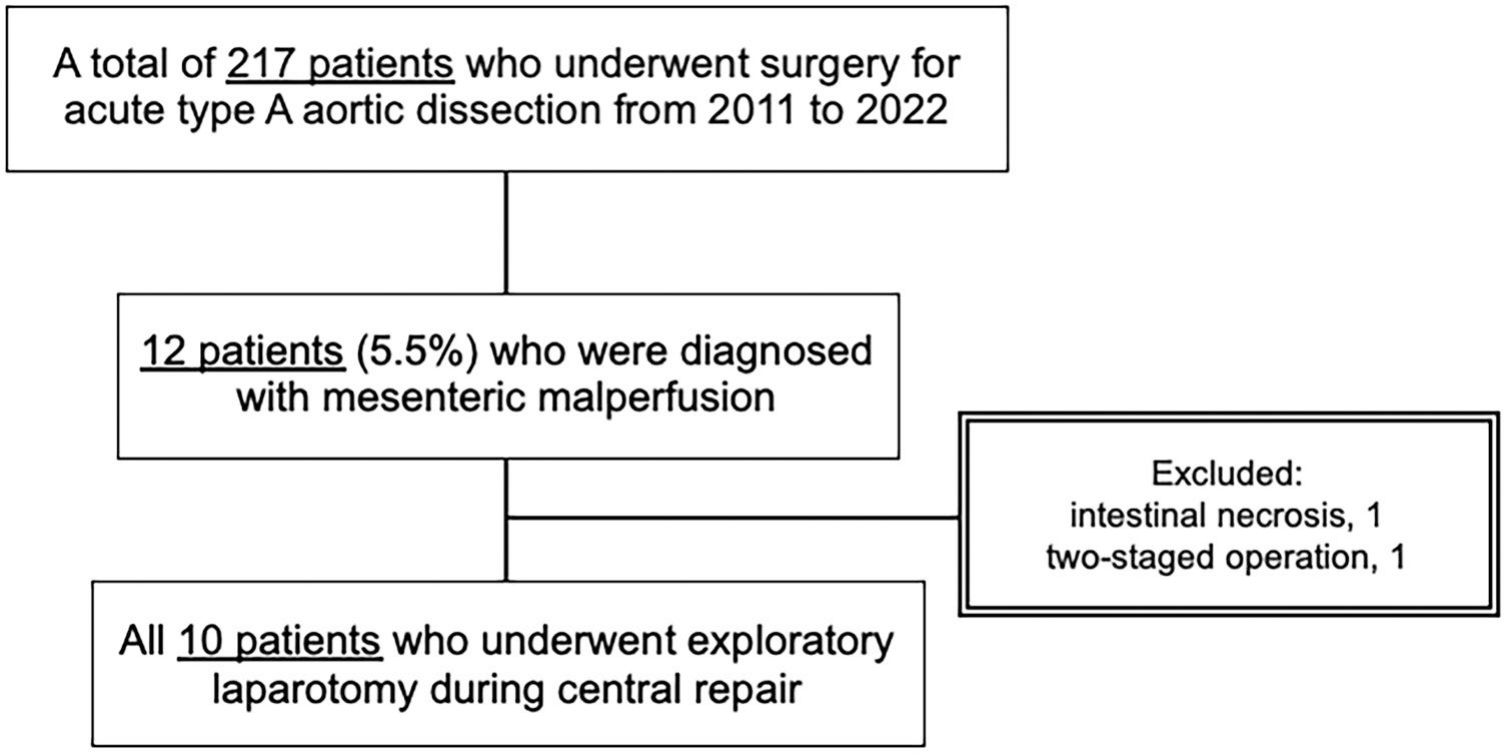
Patient selection flowchart. Mesenteric malperfusion was suspected in 12 of 217 patients with acute type A aortic dissection. The subsequent exclusion of two patients resulted in a final study cohort of 10 patients.

A total of 217 patients underwent open repair of ATAAD. The repair procedures comprised ascending aorta replacement (*n* = 110), aortic arch replacement (*n* = 98), and aortic root replacement (*n* = 9). Among these patients, 12 (5.5%) were initially suspected of having mesenteric malperfusion. Two patients were excluded based on our criteria, leaving 10 patients for analysis in this study (Fig. 3).

There were 10 patients who underwent the combined approach to repair ATAAD complicated by mesenteric malperfusion. Of the 10 patients, 6 had dynamic obstructions confirmed intraoperatively (5 by Doppler ultrasound after CPB initiation, 1 without thrombectomy). The remaining four patients had false lumen thrombosis, although it was not feasible to distinguish between purely static versus mixed obstruction. Table 1 summarizes the characteristics and surgical procedures of the study cohort.

**Table 1:** Demographic characteristics of patients with and without malperfusion

	Total (*n* = 10)	No temporary perfusion (*n* = 5)	Temporary perfusion (*n* = 5)
Age	58.5 [47.3–69.8]	70.0 [54.0–76.0]	58.0 [43.0–59.0]
Gender (M/F)	7/3	3/2	4/1
Abdominal symptoms	9 (90)	Abdominal pain, 2 Hematochezia, 1 Vomiting, 1 No symptoms, 1	Abdominal pain, 4 Hematochezia, 1
Other malperfusions	3 (30)	Lower limb, 1 BCA, 1	RCA, 1
Preoperative shock	4 (40)	2 (40)	2 (40)
Preoperative lactate level (mmol/l)	3.2 [1.8–6.5]	2.4 [1.9–4.0]	6.9 [0.89–7.2]
Operative time (min)	444 [365–539]	427 [315–460]	460 [408–570]
CPB time (min)	219 [178–268]	191 [161–194]	270 [261–293]
HCA time (min)	44 [42–58]	44 [43–53]	44 [42–62]
Cardiac arrest time (min)	113 [75–131]	102 [91–117]	133 [108–142]
Central procedure	Asc. Ao, 6 Arch, 4	Asc. Ao, 3 TAR, 2	Asc. Ao, 3 PAR, 1 TAR, 1
Intestinal resection	0 (0)	0 (0)	0 (0)
Thrombectomy	4 (40)	0 (0)	4 (80)
SMA plasty	5 (50)	0 (0)	5 (100)
Operative death Cause of death	2 (20)	1 (20) Acute myocardial infarction, 1	1 (20) Stroke, 1

Asc. Ao: ascending aorta replacement; BCA: brachiocephalic artery; CPB: cardiopulmonary bypass; HCA: hypothermic circulatory arrest; PAR: partial arch replacement; RCA: right coronary artery; SMA: superior mesenteric artery; TAR: total arch replacement.

At the time of presentation, six patients had symptoms of abdominal pain, two presented with bloody stools, and one had vomited. The remaining one patient had no complaints or abnormal abdominal findings, but the CT scan clearly showed that the SMA was occluded by dissection. Four patients (40%) were in a state of preoperative shock, defined as systolic blood pressure <80 mmHg. Of these four patients, two presented with cardiac tamponade, while the other two experienced cardiogenic shock due to either coronary artery malperfusion or acute aortic regurgitation.

All patients underwent exploratory laparotomy. During exploratory laparotomy, all patients underwent Doppler ultrasound assessment of SMA blood flow. Five patients (50%) showed reduced SMA flow and required direct perfusion via CPB. The other five patients maintained adequate SMA flow after CPB initiation and did not require any direct intervention. The time to reperfusion of the SMA was 62.0 [42.0–85.5] min from the initiation of the procedure. Our standardized surgical sequence was followed in all patients, including four patients with cardiogenic shock. In one patient with right coronary artery malperfusion, immediate CPB establishment normalized the ST changes, enabling SMA assessment during the cooling phase without delaying central repair.

The 30-day operative mortality was 20% (2/10 patients), with deaths attributed to stroke (*n* = 1) and acute myocardial infarction (*n* = 1). Postoperative contrast CT confirmed improved SMA perfusion in all surviving patients (8/10 cases). No patients required bowel resection, and no laparotomy-related complications were observed. The aortic event-free survival rate at 5 years postoperatively was 85%. Median follow-up was 8.2 years (range 1 day–12.5 years). The follow-up rate was 92.8% using the formal person-time method. During follow-up, two in-hospital deaths occurred, and one aortic event occurred at 38 months postoperatively. In the subgroup of four patients presenting with preoperative shock, one patient did not survive, yielding an operative mortality rate of 25% for this high-risk subgroup.

**Figure 4:**
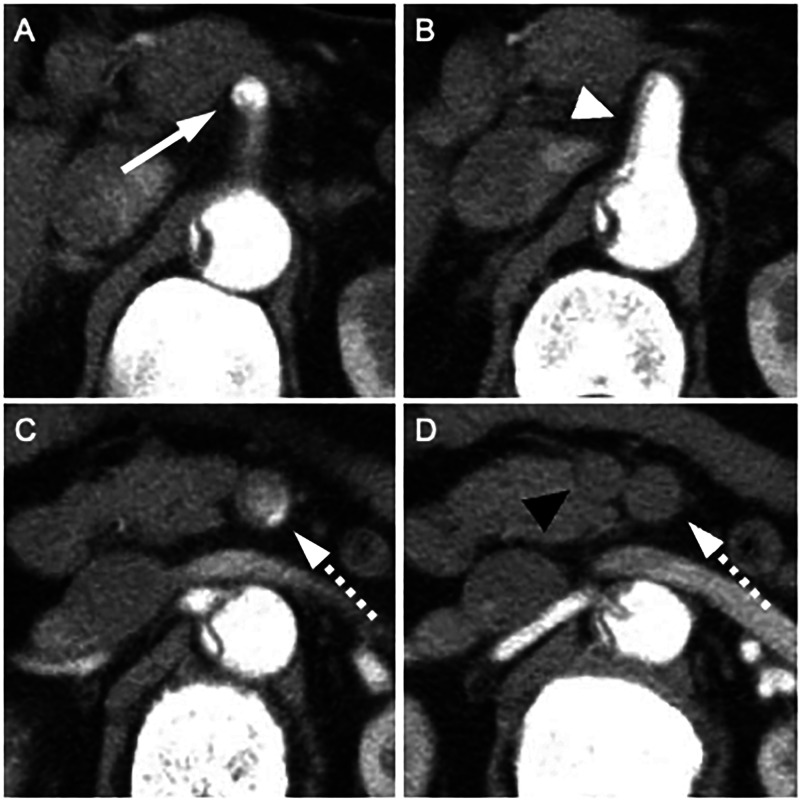
CT findings in patients with a celiacomesenteric trunk. (**A**) The celiac artery branch (white arrow) shows good contrast enhancement to the periphery. (**B**) The dissected celiacomesenteric trunk (white arrowhead) with a narrowed true lumen. (**C**) Superior mesenteric artery (SMA) branch (white dotted arrow) exhibiting poor contrast enhancement. (**D**) Peripheral occlusion of the SMA and presence of the superior mesenteric vein sign, characterized by a superior mesenteric vein diameter (black arrowhead) smaller than the SMA diameter (white dotted arrow), indicative of mesenteric ischemia.

**Figure 5:**
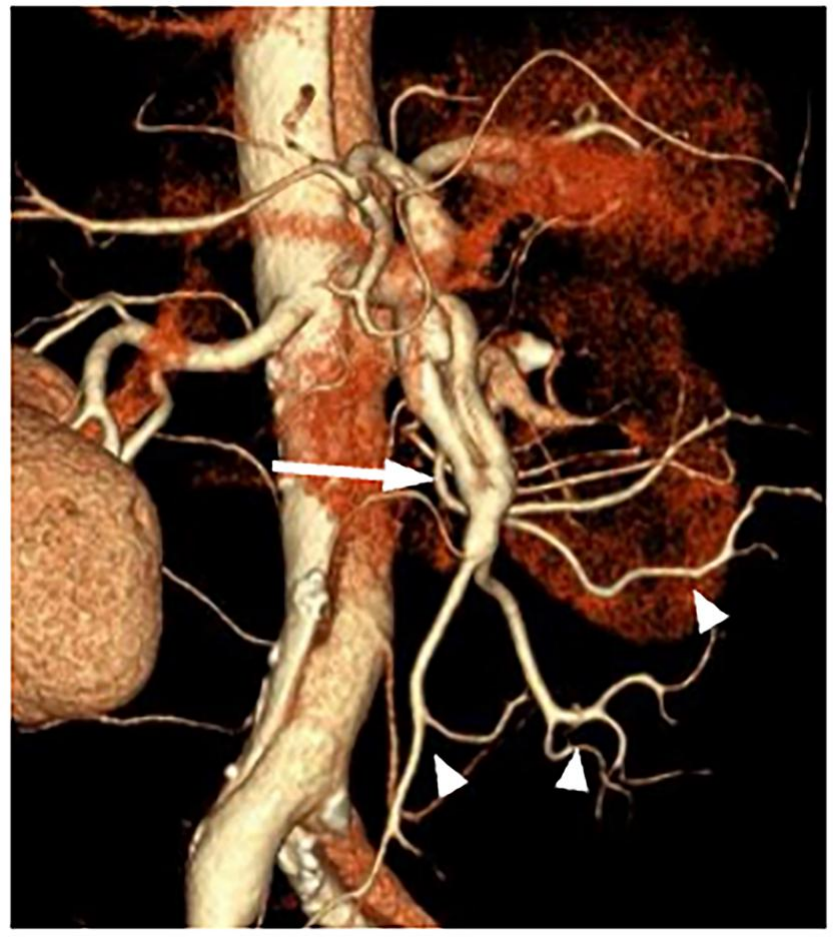
3D-CT findings 10 years after superior mesenteric artery plasty. The site of fenestration (white arrow) has well-visualized peripheral vasculature (white arrowheads), indicating long-term patency of the reconstructed SMA.

One patient had an uncommon vascular anatomical variant where both the coeliac trunk and the SMA had a common origin from the abdominal aorta as a single trunk (Fig. 4B). Dissection had progressed to its branches, but fortunately the coeliac artery branch was unaffected and there were no findings of obstruction (Fig. 4A). On imaging, the SMA demonstrated poor contrast enhancement in both the true and false lumens, consistent with dissection (Fig. 4C). Additionally, there was a reduced caliber of the superior mesenteric vein relative to the SMA, known as the smaller superior mesenteric vein sign (Fig. 4D). Temporary perfusion was therefore only performed in the SMA. After central repair, thrombectomy and SMA plasty were performed, resulting in no bowel resection and a favourable outcome. No additional interventions were required. A contrast-enhanced CT scan performed 10 years postoperatively demonstrated restored patency of the SMA with good blood flow in the previously affected segments (Fig. 5).

## DISCUSSION

Despite improvements in surgical outcomes for aortic dissection, managing malperfusion, particularly SMA malperfusion, remains a substantial challenge with high mortality rates [2, 3, 10, 11]. The critical dilemma in cases of mesenteric malperfusion secondary to ATAAD lies in determining the necessity and timing of mesenteric revascularization, complicated by the limited ischaemic tolerance of intestinal tissue.

Traditionally, central aortic repair has been prioritized, with the European Society of Cardiology (ESC) guidelines recommending immediate aortic surgery for malperfusion (Class I, Level B) [12]. However, this approach has shown suboptimal outcomes [13], as central repair alone, while potentially sufficient for purely dynamic obstructions, fails to improve blood flow in cases of static or mixed obstruction. Recent studies have shown improved efficacy with a staged approach beginning with mesenteric revascularization before addressing the proximal aortic pathology [5, 6, 14, 15]. However, this strategy is inappropriate in patients with ATAAD requiring urgent central repair due to life-threatening complications (ie, cardiac tamponade, multisystemic malperfusion, acute heart failure). Prioritizing endovascular intervention reportedly leads to mortality rates of 39% in stable patients and 100% in patients in shock [5].

While some institutions use hybrid operating rooms for simultaneous central repair and malperfusion evaluation, the ESC guidelines restrict such interventions to centers with appropriate expertise and facilities [12]. This limitation is particularly relevant in Japan, where such specialized facilities are not universally available. Our direct SMA perfusion technique offers three key advantages: concurrent revascularization during central repair, feasibility in standard operating rooms without specialized expertise, and real-time bowel perfusion assessment through laparotomy. Notably, this approach achieved remarkably improved survival outcomes—with operative mortality of 25% in shock patients and overall mortality of 20%, substantially lower than historical rates exceeding 60% in this high-risk population of ATAAD with mesenteric malperfusion. However, our small sample size without a control group prevents definitive conclusions about survival benefits. Interestingly, 50% of patients initially diagnosed with mesenteric malperfusion did not require intervention after CPB initiation. Recent studies suggest that CPB-induced haemodynamic changes may improve abdominal malperfusion [16, 17], possibly contributing to the resolution of perfusion obstruction in some cases.

Diagnosing mesenteric ischaemia in ATAAD remains challenging due to its subtle symptoms and non-specific markers [18–21]. Uchida *et al.* reported that 46% of 13 patients undergoing exploratory laparotomy showed no evidence of intestinal ischaemia [22], emphasizing the need for more accurate diagnostic methods. While Orihashi *et al.* proposed specific criteria for diagnosing mesenteric ischaemia in aortic dissection using transoesophageal echocardiography (TEE) [23], the practical application of this approach presents challenges, particularly in emergency settings. In our series, TEE was not used for SMA flow assessment because it requires advanced diagnostic expertise and carries substantial uncertainty. Given the lethal nature of bowel necrosis, we believe that it would be too risky to rely solely on TEE for decision-making.

The present study has several important limitations. First, our small sample size (*n* = 10) and retrospective design limit the generalizability of our findings and prevented meaningful statistical comparisons between groups with and without SMA perfusion. Second, the actual blood flow to the SMA during temporary perfusion was not quantified, introducing potential variability in the effectiveness of the intervention. Third, the smaller body habitus typical of the Japanese population may have allowed the 8-French tube to provide adequate perfusion, potentially limiting the applicability of these results to populations with different anthropometric characteristics.

In conclusion, while this pilot study suggests that combining exploratory laparotomy with direct perfusion may be technically feasible for addressing mesenteric malperfusion in patients with ATAAD, several considerations warrant attention. The approach carries substantial risks, including increased blood loss, potential bowel injury, prolonged operative time, additional surgical trauma, and postoperative ileus. These risks must be carefully balanced against the potential benefits of direct mesenteric reperfusion.

Larger prospective controlled studies are essential to validate these preliminary findings and establish the safety profile of this approach. Future studies should evaluate not only technical success but also long-term outcomes, potential complications, and specific patient selection criteria. Until more robust evidence is available, this approach should be considered experimental and used only in carefully selected patients.

## Data Availability

The data will be shared on reasonable request to the corresponding author.

## Notes

Presented at the 38th European Association for Cardio-Thoracic Surgery Annual Meeting, Lisbon, Portugal, 10 October 2024.

## ABBREVIATIONS

ATAAD: Acute type A aortic dissection
ESC: European Society of Cardiology
CPB: Cardiopulmonary bypass
CT: Computed tomography
SMA: Superior mesenteric artery
TEE: Transesophageal echocardiography
